# Young Hungarian Students’ Knowledge about HPV and Their Attitude Toward HPV Vaccination

**DOI:** 10.3390/vaccines5010001

**Published:** 2016-12-29

**Authors:** Bettina Claudia Balla, András Terebessy, Emese Tóth, Péter Balázs

**Affiliations:** 1 Department of Public Health, Semmelweis University, Nagyvárad tér 4., Budapest 1089, Hungary; terebessy.andras@med.semmelweis-univ.hu (A.T.); balazs.peter@med.semmelweis-univ.hu (P.B.); 2 Faculty of Medicine, Semmelweis University, Üllői út 26., Budapest 1085, Hungary; emci.toth@gmail.com

**Keywords:** HPV, HPV vaccine, cervical cancer, STD

## Abstract

(1) *Background*: Hungarys’s estimated cervical cancer mortality was 6.9/100,000 in 2012, above the average of the EU27 countries (3.7/100,000) in the same year. Since 2014, the bivalent HPV vaccine has been offered to schoolgirls aged 12–13. (2) *Methods*: We conducted a cross-sectional study among 1022 high school seniors (492 girls, 530 boys) in 19 randomly selected schools in Budapest. Our anonymous questionnaire contained 54 items: basic socio-demographic data, knowledge about HPV infection/cervical cancer and HPV vaccination. (3) *Results*: 54.9% knew that HPV caused cervical cancer, and 52.1% identified HPV as an STD. Knowledge of risk factors such as promiscuity (46.9%) and early sexual activity (15.6%) was low, but higher than that of further HPV-induced diseases: genital warts (in females 9.9%, in males 9%), anal cancer (in females 2.2%, in males 1.9%), penile cancer (9.4%), and vulvar cancer (7.8%). A percentage of 14.6% feared getting infected, and 35.7% supported compulsory HPV vaccination. A percentage of 51.2% would have their future children vaccinated—significantly more girls than boys. (4) *Conclusion*: Our results support the findings of previous studies about young adults’ HPV-related knowledge, which was poor, especially regarding pathologies in men. Despite the low level of awareness, the students’ attitude was mostly positive when asked about vaccinating their future children.

## 1. Introduction

In Hungary, the mortality rate of cervical cancer was 6.23/100,000 in 2012, which is nearly two times higher than the average (3.7/100,000) of the European Union (EU27) member countries in the same year [1]. The Hungarian national cervical cancer screening program was established in 2003 and is available free of charge for all women aged 25–65. The target population is invited to screening by traditional mail every three years. Attendance rates are relatively low; it was estimated to be around 24.3% in 2007 [2].

Currently, there are three different HPV vaccines available. All of them are recombinant and assembled from the virus-like particles (VLP) of the L1 capsid protein. While the bivalent vaccine immunizes against serotypes 16 and 18, the quadrivalent protects against LR HPV serotypes 6 and 11 as well. The 9-valent vaccine has only recently been approved by the U.S. Food and Drug Administration (FDA) in December 2014. This vaccine contains HPV types 6, 11, 16, 18, 31, 33, 45, 52, and 58. The US Centers for Disease Control and Prevention (CDC) recommends the use of all three vaccines from 9–26 years of age in both sexes [3,4].

In Hungary, 7th grade schoolgirls (12–13 years old) are offered two doses of the bivalent HPV vaccine nationwide since September 2014 [5,6]. In countries like Australia, Austria, or the USA, the vaccine is also offered to boys [7,8,9]. However, the vaccination of males has not been proved entirely cost-effective yet, except for cases when the coverage of the female population was low [10], or among men having sex with men (MSMs) [11].

Since the prevalence of HPV is the highest in the late teens and early twenties [12,13,14], the aim of our study was to analyze the knowledge of young adults about HPV-induced diseases and to assess their attitudes toward HPV vaccination. Our data of high-school seniors (18 years or above) collected in Budapest, the Hungarian capital, also provided information about the will of the respondents concerning future participation in screening programs and their receptiveness of the vaccine [15]. We adjusted our sample to the nationwide average age (17.3 years) of the first sexual intercourse [16].

## 2. Materials and Methods

Using the online database of the Educational Authority, we randomly selected 19 high schools in Budapest [17]. First, we contacted the principals via e-mail; after obtaining their approval, we visited the schools to conduct our questionnaire-based data collection between March 2013 and May 2014. We met the participating students, and their teachers typically during biology classes to present our study and give basic information about the questionnaire. We assured them that participation was voluntary and anonymous and that, after the session, we would answer any questions that occurred during the completion of the questionnaire. The questionnaire and the data sampling procedure had been approved by the board of ethics of the Semmelweis University (reference number: 32/2013). We only targeted students aged ≥18 years; therefore, parental consent was not required.

In our questionnaire, 26 matrix questions concerned basic demographic, socio-economic, and lifestyle factors, 13 questions assessed knowledge about HPV infection and cervical cancer, 11 examined the attitude toward the HPV vaccine, and 4 focused on cervical cancer screening. The overall response rate of 1277 distributed questionnaires (males 611, females 666) was 80% (males 86.7%, females 73.9%)*.* Our sample represented about 2% of the high school seniors studying in Budapest during the academic years 2012/13 and 2013/14 [18].

Individuals refusing to answer specific questions were excluded only from the evaluation of the questions concerned. Questionnaires were not processed if items concerning HPV infection and cervical cancer were left blank, or when multiple answers were given to single choice questions. We performed frequency analyses and Pearson chi-square tests—at *p* < 0.05 significance level and calculated odds ratios (ORs) with 95% confidence intervals—using IBM-SPSS v.23 (IBM Magyarországi Kft. H-1117 Budapest, Infopark, Neumann János u. 1., Hungary).

## 3. Results

### 3.1. Socio-Demographic Background and Lifestyle Factors

Our sample of high school seniors (*N* = 1022) consisted of 492 female (48.1%) and 530 male (51.9%) students. They studied in vocational (65.4%) and grammar schools (34.6%). The majority (57.4%) wanted to pursue education after high school graduation. 31.5% of mothers and 25.1% of fathers had undergraduate or graduate degrees. Percentages of 11.4% of mothers and 1.6% of fathers were healthcare providers. The majority of students (82.8%) perceived their family’s economic status as up to the national average or above it. A percentage of 25% admitted to being affiliated with religious organizations. A percentage of 75% didn’t follow any specific diet, 36.9% regularly practiced some kind of physical activity, and 89.9% used the Internet for at least 1 h a day.

### 3.2. Knowledge about HPV Infection

#### 3.2.1. Prevalence Analysis

The majority (64.4%) knew that HPV caused cervical cancer; however, they were less aware of other related diseases. Genital warts were recognized by less than 10% (in females 9.9%, in males 9%) and anal cancer by less than 3% (in females 2.6%, in males 1.9%). A percentage of 21.5% falsely believed that HPV could cause female infertility, and 14.5% were not aware of HPV-induced pathologies in men. A percentage of 13.7% considered the virus as responsible for prostate cancer. “I don’t know” answers were relatively frequent, especially for questions concerning pathologies in men (59%). Table 1 shows pathologies associated with HPV infection (*N* = 1022). 

Concerning the ways of transmission (Table 2), the majority (52.1%) identified HPV infection as a sexually transmitted disease (STD). Vertical (mother-to-child) transmission and skin contact, however, were only recognized by 10.5% and 3.1%. More than one-third of the sample (36.4%) had no information about the mode of transmission of the disease (*N* = 1022).

Nearly half of the sample (46.9%) knew that promiscuity was a risk factor for HPV infection, and 41.7% considered unprotected sex to be a risk factor (Table 3). Early initiation of sexual life was indicated by 15.6% of the students (*N* = 1022).

The majority (66.7%) knew that cervical cancer screening and HPV vaccination (58.3%) were means of prevention (Table 4).

Considering the information sources of HPV infection/cervical cancer (Table 5), students gained knowledge from the Internet (23.9%), from family members and friends (23.1%), and through traditional channels of mass communication (TV, radio) (20.5%).

#### 3.2.2. Gender-Related Comparisons

While exploring associations and ORs (Table 6), we compared the knowledge of female and male students. Both sexes were almost equally represented (*n* = 492 vs. *n* = 530). More female than male students knew that HPV could cause cervical cancer (*p* < 0.001, OR = 4.1, 95% CI: 3.08–5.46) and genital warts in women (*p* < 0.05, OR = 1.7, 95% CI: 1.13–2.60). More females than males believed that HPV could cause infertility (*p* < 0.001, OR = 1.93, 95% CI: 1.43–2.62). As for spreading the infection, more females than males knew that HPV was an STD (*p* < 0.001, OR = 4.3, 95% CI: 3.31–5.59). Table 6 shows only significant associations.

Table 7 shows significant differences between the knowledge of female and male students about common risk factors for HPV infection and cervical cancer. Females had significantly more knowledge than males.

### 3.3. Attitude Toward the HPV Vaccine

Compulsory vaccination was supported by 91.2%, and 58.0% believed it was important. The majority (73.2%) had heard about the HPV vaccine, 16.1% admitted to having already received it, and a further 10.7% wished to be vaccinated in the future. While exploring the motivations behind accepting or rejecting the vaccine, we found that only 14.6% realized their own risk of getting infected by the virus. When asked about the efficacy of the vaccine, 58.7% trusted it with some doubts, while 24.3% had no doubts at all. A percentage of 35.7% of the sample preferred compulsory vaccination, and the slight majority of students (51.2%) would have the vaccine administered to their future children (Table 8).

Gender-related attitudes are demonstrated in Table 9. Outcomes of all variables were significant. More females responded with “yes” answers, except for the question regarding the families’ positive attitude toward mandatory vaccination in general.

We also explored the difference between the attitudes of vaccinated and unvaccinated students. Those who had already been immunized or opted to be vaccinated in the future had significantly more positive attitudes than the unvaccinated subsample. Vaccinated students were more likely to have families supportive of mandatory vaccination (Table 10).

## 4. Discussion

According to previous international studies, disease awareness has a positive impact on the attitude of young adults toward HPV vaccination; thus, it must be considered as a protective factor [19,20,21]. There was also a positive association between knowledge, trust in the vaccine, and the number of received doses, which indicated that more trust in the vaccine resulted in higher acceptance [20,22]. These findings emphasize the important role of health education in improving the vaccine coverage of the target population.

Our sample of high school seniors in Budapest had relatively poor knowledge about HPV infection and cervical cancer. While the majority (64.4%) was familiar with the etiology of cervical cancer, they were not aware of other pathologies caused by the virus in the anogenital region: less than 10% linked the virus to genital warts, less than 3% to anal cancer, and 9.4% to penile cancer.

The knowledge about risk factors for HPV infection was also low—only promiscuity and unprotected sex were recognized as risk factors by over 40% of the sample. Concerning the ways of transmission, 52.1% knew that the disease could be mediated by sexual intercourse; skin contact was only identified by 3.1%. An alarmingly low number of students (14.6%), albeit significantly more girls than boys, recognized their personal risk of infection. In terms of prevention, around two-thirds of the sample knew about cervical cancer screening (66.7%) and HPV vaccination (58.3%). Finally, it must be stressed that the number of “I don’t know” answers was relatively high for questions assessing the knowledge and attitude of the sample.

The students’ source of information was diverse: 23.9% obtained their knowledge from the Internet, 23.1% from family members and friends, and 20.5% from TV/radio. Surprisingly, healthcare professionals played only a marginal role in providing information about the disease. The most frequently consulted providers were gynecologists (13.1%), while GPs and registered nurses represented nearly the same proportions (11.4% and 10.5%). This low representation may be explained by the specific age and health status of the target population. Young adults are generally healthier; therefore, they have less contact with members of the medical staff than the ageing population. It must be emphasized that the students gained information typically from online platforms, which underlines the role and responsibility of public service and social media in health education.

Female students had significantly higher level of knowledge than males. Since cervical cancer is a pathology affecting only women—targeted by a national screening and vaccination program—it is understandable that females have more knowledge about the disease and its causative agent (HPV). Furthermore, school-based HPV-specific sexual education programs tend to focus on girls rather than boys. It must also be considered that girls, when entering procreative age and starting sexual life, usually visit a gynecologist who provides them with information about HPV infection and cervical cancer. Nevertheless, analyzing the deeper knowledge of young women concerning health issues should be the subject of further investigations.

In Hungary, the vaccine was licensed in 2007 but was only available on private financing. Since September 2014, the bivalent HPV vaccine is administered free of charge among schoolgirls aged 12–13 (7th graders) as part of the public vaccination program. Our research was conducted prior to this date (March 2013–May 2014); thus, it provides baseline data for future comparative studies.

The majority of the students in our sample had already heard about the HPV vaccine (73.2%). Significantly more male students reported that their families supported mandatory vaccination; nevertheless, significantly more females than males were informed about the HPV vaccine. Almost 80% of the sample believed in its efficacy, although around 60% had doubts, despite their positive attitudes. Significantly more females than males trusted the efficacy of the vaccine. A percentage of 16.1% reported to having already been vaccinated, and an additional 10.5% opted for it in the future. More than half of the sample (51.2%)—significantly more females than males—would have their future children vaccinated. Compulsory vaccination of HPV was supported by 35.7% of the sample (significantly more females than males); however, 32.9% rejected it, while the rest of the students remained undecided.

Students already vaccinated or wishing to be vaccinated in the future were more supportive of immunization against HPV than the rest of the sample. This finding is consistent with the results of previous studies [23]. Female students were also more likely to have a positive attitude toward the HPV vaccine, which can be explained by their more thorough knowledge about HPV and cervical cancer. Self-perceived risk of HPV infection and the level of trust in the efficacy of the vaccine were also significantly higher among girls, both attributable to the gender-related discrepancy of attitudes. This inequality concerning knowledge and attitudes has already been reported by previous international studies, as well as the generally poor knowledge of young adults about the disease [24,25,26,27,28]. The relatively high receptiveness of the HPV vaccine, despite the low level of knowledge, has also been reported [29,30].

In certain countries (Austria, Australia, and the USA), the vaccine is offered to both males and females; nevertheless, its cost-effectiveness among the male population is still debated [31,32,33,34]. Currently, the introduction of free HPV vaccines for men is not on the agenda of the Hungarian healthcare system.

## 5. Conclusions

Due to the low level of knowledge of high school seniors, health and sexual education of young adults should be reconsidered in Hungary, especially for the male population. It should be emphasized that HPV infection is sexually transmitted; thus, both sexes are affected, and awareness of the disease is a protective factor. Despite their lack of knowledge and low self-perceived risk, the students mostly supported the HPV vaccination program; however, higher vaccine acceptance and better vaccination coverage could be achieved by the implementation of more tailored health and sex education programs. We have been unaware of the existence of any similar nationwide campaigns in Hungary since data collection began.

## Figures and Tables

**Table 1:** Distribution of answers concerning pathologies associated with HPV infection.

Pathology	In Females	In Males
vulvar cancer	7.8%	
*infertility*	*21.5%*	
cervical cancer	64.4%	
penile cancer		9.4%
*prostate cancer*		*13.7%*
*no pathology at all*		*14.5%*
head-neck cancer	2.2%	1%
anal cancer	2.6%	1.9%
genital warts	9.9%	9%
“I don’t know”	29.7%	59%

Note: Answers in italic are considered to be false.

**Table 2:** Distribution of answers concerning the mode of transmission of HPV infection.

Mode of Transmission	%
STD	52.1%
*blood and saliva*	20%
*droplet contact*	13.8%
vertical	10.5%
skin contact	3.1%
*fecal-oral*	2.1%
“I don’t know”	36.4%

Note: Answers in italic are considered to be false.

**Table 3:** Distribution of answers concerning risk factors for HPV infection.

Risk Factor	%
promiscuity	46.9%
unprotected sex	41.7%
promiscuous partner	30.6%
early initiation of sexual life	15.6%
“I don’t know”	35.3%

**Table 4:** Distribution of answers considering the means of cervical cancer prevention.

Means of Prevention	%
cervical cancer screening	66.7%
HPV vaccine	58.3%
safe sex	31.5%

**Table 5:** The students’ sources of information about HPV infection and cervical cancer.

Source	%
Internet	23.9%
family and friends	23.1%
TV, radio	20.5%
other	14.2%
gynecologist	13.1%
GP	11.4%
specialist nurse	10.5%
print media, books	3.77%
other healthcare worker	6.2%

**Table 6:** Gender-based comparison of knowledge about the etiology and spreading of HPV infection.

Variables	Males *n* (%)		Females *n* (%)		*p*-Value	OR	CI95
	no	yes	no	yes			
Can HPV cause cervical cancer?	252 (47.5%)	278 (52.5%)	89 (18.1%)	403 (81.9%)	< 0.001	4.10	3.08–5.46
Can HPV cause genital warts in women?	489 (92.3%)	41 (7.7%)	430 (87.4%)	62 (12.6%)	< 0.05	1.72	1.13–2.60
Can HPV cause infertility?	442 (83.4%)	88 (16.6%)	355 (72.2%)	137 (27.8%)	< 0.001	1.93	1.43–2.62
Can HPV be transmitted sexually?	343 (64.7%)	187 (35.3%)	147 (29.9%)	345 (70.1%)	< 0.001	4.30	3.31–5.59

**Table 7:** Gender-based comparison of knowledge about common risk factors for HPV infection and cervical cancer.

Risk Factors		Males *n* (%)	Females *n* (%)	*p*-Value	OR	CI95
early initiation of sexual life	HPV infection	62 (11.7%)	97 (19.7%)	<0.001	1.85	1.31–2.61
	cervical cancer	75 (14.1%)	108 (21.9%)	<0.001	1.70	1.23–2.36
promiscuity	HPV infection	182 (34.3%)	297 (60.3%)	<0.001	2.91	2.25–3.75
	cervical cancer	196 (36.9%)	281 (57.1%)	<0.001	2.26	1.17–2.91
partners’ promiscuity	HPV infection	119 (22.4%)	194 (39.4%)	<0.001	2.24	1.71–2.95
unprotected sex	HPV infection	162 (30.6%)	264 (53.7%)	<0.001	2.63	2.03–3.39
	cervical cancer	119 (22.5%)	269 (54.7%)	<0.001	4.16	3.17–5.46

**Table 8:** Knowledge about/attitudes toward HPV vaccination.

**Variables**	**Yes (%)**	**No (%)**	**Undecided (%)**
Heard about the HPV vaccine	73.2	26.4	-
Has a vaccinated family member	15.2	41.8	-
Considers to be at risk of getting infected by HPV	14.6	45.0	-
**Variables**	**Yes (%)**	**No (%)**	**Undecided (%)**
Would vaccinate his/her future children	51.2	22.8	26.1
Would make the vaccine compulsory	35.7	32.9	31.4
**Variables**	**Yes (%)**	**No (%)**	**Yes, but with doubts (%)**
Believes in the efficacy of the vaccine	24.3	17	58.7
**Variables**	**Yes (%)**	**No (%)**	**No, but would opt for it (%)**
Has already received the HPV vaccine	16.1	73.1	10.7

**Table 9:** Gender-based attitudes toward HPV vaccination.

Variables	Males *n* (%)		Females *n* (%)		*p*-Value
	Yes	No	Yes	No	
Family favors all mandatory vaccination	106 (25.4%)	311 (74.6%)	74 (16.4%)	377 (83.6%)	<0.001
Has already received the HPV vaccine	27 (7.6%)	326 (92.4%)	101 (22.9%)	339 (77.1%)	<0.001
Considers to be at risk of getting infected by HPV	39 (17.2%)	187 (82.8%)	90 (29.9%)	211 (70.1%)	<0.001
Would vaccinate his/her future children	201 (59.2%)	138 (40.8%)	260 (79.5%)	67 (20.5%)	<0.001
Believes in the efficacy of the vaccine	307 (79%)	82 (21%)	377 (86.7%)	58 (13.3%)	<0.001
Would make the vaccine compulsory	114 (41.8%)	159 (58.2%)	211 (60%)	141 (40%)	<0.001

**Table 10:** Attitudes toward HPV vaccination based on vaccination status.

Variables		Males Vaccinated/Opting for/Non-Vaccinated (%)	Females Vaccinated/Opting for/Non-Vaccinated (%)	*p*-Value
Family favors all mandatory vaccination	yes	10/7/56 (13.7/9.6/76.7)	19/16/32 (28.3/24/47.7)	<0.001
	no	15/14/225 (5.9/5.5/88.6)	76/45/236 (21.2/12.7/66.1)	
Considers to be at risk of getting infected by HPV	yes	8/4/19 (25.8/12.9/61.3)	17/17/52 (19.8/19.8/60.4)	<0.001
	no	11/8/147 (6.6/4.8/88.6)	57/21/127 (27.8/10.2/62)	
Would vaccinate his/her future children	yes	21/18/107 (14.4/12.3/73.3)	93/51/103 (37.7/20.6/41.7)	<0.001
	no	4/5/111 (3.3/4.2/92.5)	1/2/63 (1.5/3/95.5)	
Believes in the efficacy of the vaccine	yes	21/12/190 (9.4/5.4/85.2)	95/55/201 (27/15.7/57.3)	<0.001
	no	3/6/60 (4.3/8.7/87)	1/2/49 (1.9/3.9/94.2)	
Would make the vaccine compulsory	yes	17/12/55 (20.2/14.3/65.5)	64/48/92 (31.4/23.5/45.1)	<0.001
	no	8/5/125 (5.8/3.6/90.6)	20/6/109 (14.8/4.5/80.7)	
